# Soluble Epoxide Hydrolase Inhibitors Regulate Ischemic Arrhythmia by Targeting MicroRNA-1

**DOI:** 10.3389/fphys.2021.717119

**Published:** 2021-09-27

**Authors:** Yanying Chen, Qiong Liu, Tian Yang, Li Shen, Danyan Xu

**Affiliations:** Department of Internal Cardiovascular Medicine, Second Xiangya Hospital, Central South University, Changsha, China

**Keywords:** miR-1, EETs, t-AUCB, SRF, PI3K/Akt/GSK3β pathway

## Abstract

**Background:** Soluble epoxide hydrolase inhibitors (sEHis) inhibit the degradation of epoxyeicosatrienoic acids (EETs) in cells, and EETs have antiarrhythmic effects. Our previous experiments confirmed that t-AUCB, a preparation of sEHis, inhibited ischemic arrhythmia by negatively regulating microRNA-1 (miR-1), but its specific mechanism remained unclear.

**Aim:** This study aimed to examine the role of serum response factor (SRF) and the PI3K/Akt/GSK3β pathway in t-AUCB-mediated regulation of miR-1 and the interaction between them.

**Methods/Results:** We used SRF small interfering RNA (siSRF), SRF small hairpin (shSRF) RNA sequence adenovirus, PI3K/Akt/GSK3β pathway inhibitors, t-AUCB, and 14,15-EEZE (a preparation of EETs antagonists) to treat mouse cardiomyocytes overexpressing miR-1 and mice with myocardial infarction (MI). We found that silencing SRF attenuated the effects on miR-1 and its target genes KCNJ2 and GJA1 in the presence of t-AUCB, and inhibition of the PI3K/Akt/GSK3β pathway antagonized the effects of t-AUCB on miR-1, KCNJ2, and GJA1, which were associated with PI3Kα, Akt, and Gsk3β but not PI3Kβ or PI3Kγ. Moreover, the PI3K/Akt/GSK3β pathway was involved in the regulation of SRF by t-AUCB, and silencing SRF inhibited the t-AUCB-induced increases in Akt and Gsk3β phosphorylation.

**Conclusions:** Both the SRF and the PI3K/Akt/GSK3β pathway are involved in the t-AUCB-mediated regulation of miR-1, and these factors interact with each other.

## Introduction

Recently, the incidence and mortality of coronary heart disease have increased each year. Malignant arrhythmia caused by ischemic cardiomyopathy is the main reason for sudden cardiac death (Peters et al., 2019). Traditional antiarrhythmic drugs cannot completely treat lethal ischemic arrhythmia; thus, finding new antiarrhythmic targets and drugs is a difficult and hot topic in modern antiarrhythmic research.

Epoxyeicosatrienoic acids (EETs) are a class of endogenous lipid epoxides with strong biological activity. Epoxyeicosatrienoic acids significantly dilate blood vessels, inhibit inflammation and platelet aggregation, inhibit the migration and proliferation of vascular smooth muscle cells, promote angiogenesis (Yang et al., 2009; Nithipatikom and Gross, 2010), and regulate lipid metabolism and insulin resistance (Newman et al., 2005; Skepner et al., 2011). EETs have a short intracellular half-life and can be quickly catalytically degraded by soluble epoxide hydrolase (sEH) (Spector et al., 2004); therefore, the application of soluble epoxide hydrolase inhibitors (sEHis) is an effective way to increase the concentration and utility of intracellular EETs (Inceoglu et al., 2007; Guo et al., 2018). Our previous studies showed that sEHis could significantly inhibit the occurrence of malignant arrhythmias, such as ventricular tachycardia and ventricular fibrillation in mice with cardiac hypertrophy (Xu et al., 2006), but the mechanism was unclear.

Studies have shown that microRNA-1 (miR-1) has arrhythmogenic effects, and myocardial-specific miR-1 overexpression can induce arrhythmia in the context of myocardial ischemia and hypoxia (Yang et al., 2007; Terentyev et al., 2009; Cheng et al., 2010; Wang et al., 2010). Researchers have shown that the injection of miR-1 into the ischemic myocardium of rats with myocardial infarction (MI) led to decreases in GJA1 and KCNJ2, which are the target genes of miR-1. As a result, the expression of connexin 43 and Kir2.1, which are encoded by GJA1 and KCNJ2, respectively, was significantly reduced. Connexin 43 slowed electrical conduction, while Kir2.1 reduced the inward rectifier potassium current (I_K1_), and these changes slowed the conduction rate, led to the formation of a pathological turn-back circuit, and increased internal calcium flow, which caused cell membrane electrical potential oscillation (after depolarization) and ultimately led to arrhythmia. However, researchers also found that injecting miR-1-specific 2′-O-methyl-modified antisense oligoribonucleotide, a specific miR-1 inhibitor, could significantly improve these changes (Yang et al., 2007). On this basis, our previous studies further confirmed *in vitro* and *in vivo* that sEHis protected against ischemic arrhythmia through negative regulation of miR-1 (Liu et al., 2017), but the specific regulatory mechanism was unknown.

Serum response factor (SRF) is a highly conserved and widely expressed transcription factor (Norman et al., 1988). SRF is a gene that regulates the growth and development of many organs by regulating the cytoskeleton, transcription factors, and cell signals (Olson and Nordheim, 2010). The expression of miR-1 and SRF was increased during hyperglycemia, and after the knockdown of SRF, the transcription and expression of miR-1 were inhibited, suggesting that miR-1 was positively regulated by SRF (Shan et al., 2010). On the other hand, Lu et al. (2009) believed that SRF was closely related to ischemic arrhythmia, and Fernandez-Sada et al. (2017) suggested that SRF was significantly elevated in rats with metabolic syndrome. When healthy rat cardiomyocytes were exposed to the serum of diseased rats, they showed impaired contractile function and Ca^2+^ treatment. This suggested that SRF might play an important role in arrhythmia events. Moreover, our previous study demonstrated that sEHi might regulate SRF (Gui et al., 2018). According to the above results, we speculated that SRF might participate in the regulation of sEHi on miR-1 and was closely related to the anti-ischemic arrhythmic effect of sEHi. This idea is worthy of further verification.

PI3K is closely related to arrhythmic caused by ischemia. A study showed that the administration of a PI3K inhibitor to canine cardiomyocytes could prolong the myocardial action potential repolarization time course (action potential duration, APD) in a concentration-dependent manner. On this basis, the perfusion of PIP3, which is the second messenger produced by PI3K, could completely reverse these effects, suggesting that inhibiting PI3K activity might cause arrhythmia (Lu et al., 2012). In addition, Gong et al. (2017) showed that microRNA-mediated activation of the PI3K pathway could attenuate myocardial cell damage caused by ischemia and hypoxia. At present, studies have shown that PI3K and SRF can interact (Chang et al., 2004; Lien et al., 2006), but the relationship between these factors is still unclear in the context of inhibiting ischemic arrhythmia through the negative regulation of miR-1 by sEHis. The purpose of this study was to investigate the roles of SRF and the PI3K/Akt/GSK3β pathway and their interaction in the negative regulation of miR-1 by t-AUCB.

## Methods and Materials

### Main Reagents

**Table:** 

**Primary reagent name**	**Manufacturer**
SRF primary antibody	Cell signaling technology
siSRF	Thermo Fisher Scientific
HS173	Selleck
Wortmannin	Selleck
TGX221	Selleck
AS252424	Selleck
MK2206 2HCL	Selleck
TWS119	Selleck
Akt	Cell signaling technology
Gsk3β	Cell signaling technology

### Preparation of t-AUCB

For the *in vitro* experiments, 41.25 mg of t-AUCB powder (a gift from Bruce D Hammock, University of California, Davis, CA, USA) was weighed and dissolved in 500 μl of dimethyl sulfoxide (DMSO) to prepare a stock solution with a final concentration of 0.2 M. For the *in vivo* experiments, we added 50 mg of t-AUCB dry powder to 1,000 ml of distilled water and mixed it with ultrasonication for 1 h until the powder was fully dissolved to prepare a 50 mg/L t-AUCB stock solution. Both solutions were stored at room temperature. In our previous study, we verified that 5 mg/L t-AUCB and 20 μM t-AUCB were the best doses to treat mice with MI and primary cultured neonatal mouse cardiomyocytes, respectively, thus, in our study, we used 5 mg/L t-AUCB and 20 μM t-AUCB as treatments.

### Mice and Mouse Model of MI

Eight-week-old male Kunming mice were purchased from Hunan Slack Jingda Experimental Animal Co., Ltd. The mice were anesthetized, and then the left anterior descending coronary artery was ligated to establish an MI model. The sham group underwent thoracic surgery without coronary artery occlusion.

### Electrophysiological Studies

Electrophysiological studies were performed as previously described (Xu et al., 2006). The mice were awake. The negative electrode was connected to the left forefoot skin, and the positive electrode was in close contact with the corresponding back skin. The positive electrode of the ECG monitor was connected to the left lower limb, the negative electrode was connected to the right forelimb, and the ground wire was connected to the right lower limb. The ECG signals of lead II were continuously monitored and recorded. The duration of pulsed electric field stimulation was 1 min, the frequency of pulsed electric field stimulation was 10 Hz, the voltage of pulse electric field stimulation was 30 V, and the duration of ECG observation was 0–60 min.

### Immunohistochemistry

The hearts were fixed with 10% buffered formalin for 24–48 h and paraffin embedded, and then the samples were sectioned into 4-μm slices. After deparaffinization, the samples were stained with hematoxylin-eosin (HE) for histological examination. The area of infarction was measured using ImageJ and expressed as a percentage of the total left ventricular area.

### Gene Transfection

Neonatal mice were selected 1 day after birth, and primary mouse ventricular myocytes were cultured for 24 h. Then, effective SRF small interfering RNA (siSRF) sequences and micrONTM mmu-miR-1a-3p croRNA-1 (agomiR-1), an agonist of miR-1, were constructed and cotransfected into cells for 24 h to successfully establish an SRF-silencing cell model and an miR-1 overexpression (agomiR-1) model, respectively. Based on the *in vitro* experiments, the mice were treated by local myocardial injection (Davideit et al., 2019) of effective SRF small hairpin (shSRF) RNA sequence adenovirus and negative control (Neg Ctl) adenovirus to establish a mouse model of myocardial SRF silencing.

### Real-Time Quantitative PCR

RNA extraction and real-time quantitative PCR were performed as previously described (Guo et al., 2018). The primer sequences were as follows, and data were normalized to GAPDH and expressed as a relative ratio.

**Table:** 

**Genes**	**Sequences (5^**′**^-3^**′**^)**
**miR-1**	
Forward	TCAATCTCTAACAAGCTAATCTCT
Reverse	TTGACAGTAGGTTAATCCAAAGT
**SRF**	
Forward	AGCAAGCGTCTCCCTCTC
Reverse	GGGGACTAGGGTACATCA
**KCNJ2**	
Forward	GGAATGGCAAGAGTAAAGTCCA
Reverse	AGGGCTATCAACCAAAACACA
**GJA1**	
Forward	CTTGGGGTGATGAACAGT
Reverse	TGAGCCAAGTACAGGAGT
**GAPDH**	
Forward	AAGCCCATCACCATCTTCCAGGAG
Reverse	AGCCCTTCCACAATGCCAAAG

### Western Blotting

Myocyte protein concentrations were determined by the bicinchoninic acid (BCA) method. Proteins were fractionated on 10% sodium dodecyl sulfate (SDS)-polyacrylamide gels and transferred to polyvinylidene difluoride (PVDF) membranes. Western blotting was performed as the standard method (Guo et al., 2018). Western blotting was performed with rabbit anti-mouse SRF, Akt, p-Akt, GSK3β, and p-GSK3β antibodies (Cell Signaling Technology, Danvers, MA, USA), and β-actin was used as a loading control. ImageJ (NIH, Bethesda, MD, USA) software was used to quantify the pixel intensities of immunoreactive bands.

### Data and Statistical Analysis

All the data were analyzed using the SPSS 16.0 software package. All test indicators were tested for normality, and each measurement data point was expressed as the mean ± standard error (SEM). Independent sample *t*-tests were used to compare the mean between the two groups, and one-way ANOVA was used to compare multiple samples. The main experimental data came from more than three repeated experiments, two-sided test *P* < 0.05, the difference was considered statistically significant.

## Results

### The Role of SRF in the Cardioprotective Effect of t-AUCB

In our previous study, we found that t-AUCB (a sEHi) negatively regulated miR-1. In this study, we examined that SRF participated in the protective effects of t-AUCB in MI hearts. In the past, we also found that the expression of SRF in the myocardial ischemic area in mice was significantly decreased compared with that in the control, while the expression of miR-1 was robustly increased, and t-AUCB significantly inhibited these changes in mice with MI.

**Figure 1:**
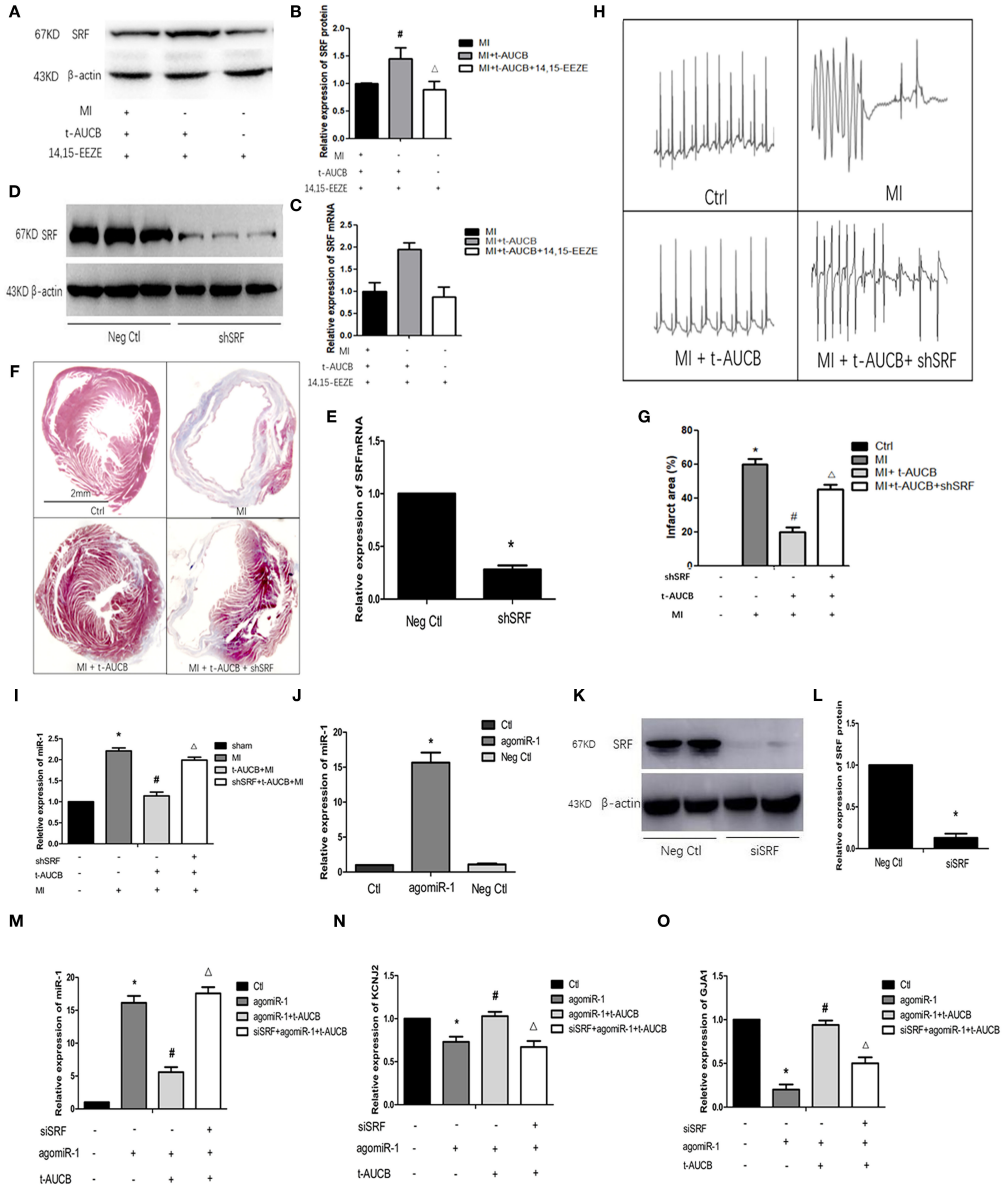
T-AUCB negatively regulates miR-1 through SRF. MicrONTM mmu-miR-1a-3p croRNA-1 (agomiR-1), serum response factor (SRF), small interfering RNA (siSRF), and t-AUCB were used to treat mouse cardiomyocytes, and then SRF, microRNA-1 (miR-1), and its target genes were detected. On the other hand, SRF small hairpin (shSRF) RNA sequence adenovirus, t-AUCB, and 14,15-EEZE were used to treat mice with myocardial infarction (MI), and the changes of MI area, ECG, SRF, and miR-1 were checked. **(A)** Grayscale images of SRF after 14,15-EEZE intervention (*n* = 4). **(B)** Relative protein expressions of SRF (*n* = 4). **(C)** Relative mRNA expressions of SRF (*n* = 4). **(D)** Protein bands of SRF silencing mouse model (*n* = 4). **(E)** Relative mRNA expressions of SRF silencing mouse model (*n* = 4). **(F)** SRF participated in t-AUCB-mediated regulation of MI areas in MI mice (*n* = 3). **(G)** Infarct size expressed as percentage of left ventricular area for each group (*n* = 3). **(H)** SRF was involved in the antiarrhythmic effect of t-AUCB (*n* = 3). **(I)** T-AUCB reduced miR-1 in ischemic area, which could be reversed by shSRF (*n* = 5). **(J)** Construction of agomiR-1 model (*n* = 4). **(K)** Construction of SRF silencing cell model (*n* = 4). **(L)** Relative protein expressions of SRF silencing model (*n* = 4). **(M)** SiSRF reversed the negative regulation of miR-1 by t-AUCB in cells (*n* = 4). **(N)** Silencing SRF antagonized the effect of t-AUCB on KCNJ2 (*n* = 4). **(O)** Silencing SRF antagonized the effect of t-AUCB on GJA1 (*n* = 4). Compared with the control (Ctl) group, *P < 0.05; compared with the agomiR-1 group, ^#^*P* and *P < 0.05; compared with the agomiR-1 + t-AUCB group, ^Δ^*P* and *P < 0.05.

To verify that SRF was directly regulated by EETs, we examined the relationship between t-AUCB and SRF expression in cultured neonatal cardiomyocytes. We observed that t-AUCB upregulated SRF mRNA by 1.94-fold and SRF protein by 1.47-fold, while 14,15-EEZE (2.5 mg/kg), a preparation of EET antagonists repressed these changes (Figures 1A–C, *n* = 4, *P* < 0.05), and these data suggested that sEHis positively regulated the expression of SRF.

To further confirm the role of SRF in the antiarrhythmic effect of sEHis, we injected shSRF into the myocardium of mice with MI and verified the effect of silencing (Figures 1D,E, *n* = 4, *P* < 0.05). Furthermore, we measured the infarction area, and the results are shown in Figure 1. Compared with that in the MI group, the myocardial infarct size decreased from 60 to 20% in t-AUCB-treated mice with MI, and the infarct size increased from 20 to 45% when the mice were treated with t-AUCB and shSRF together (Figures 1F,G, *n* = 3, *P* < 0.05). Moreover, we found that there was no arrhythmia in either the control group or MI+ t-AUCB group, when the mice were stimulated with 30 V electrical stimulation, while ventricular tachycardia was observed in the MI group and mice with MI that were treated with both t-AUCB and shSRF when they were stimulated with the same electrical stimulation (Figure 1H, *n* = 3, *P* < 0.05). This result suggested that SRF silencing could block the effect of t-AUCB and lead to arrhythmia.

Thus, we silenced SRF in mice with MI and found that the expression of miR-1 in the myocardial ischemic area was significantly increased to 2.21-fold compared with that in the control group and reduced to 51% after the t-AUCB intervention compared with that in mice with MI; however, SRF silencing reversed the effects of t-AUCB. We observed that miR-1 in the shSRF+ t-AUCB + MI group increased 1.75-fold compared with that in the t-AUCB + MI group (Figure 1I, *n* = 5, *P* < 0.05). To further verify the relationship between SRF and miR-1, we successfully constructed an agomiR-1 model in neonatal mouse cardiomyocytes (Figure 1J, *n* = 4, *P* < 0.05). After silencing SRF (Figures 1K,L, *n* = 4, *P* < 0.05), we measured the expression of miR-1 and its target genes KCNJ2 and GJA1. We found that compared with those in the agomiR-1 group, miR-1 decreased and its target genes increased by t-AUCB in the agomiR-1 + t-AUCB group, and siSRF increased miR-1 to 3.14-fold and decreased KCNJ2 and GJA1 mRNA to 66 and 54%, respectively (Figures 1M–O, *n* = 4, *P* < 0.05) compared with those in the agomiR-1 + t-AUCB group. Therefore, SRF played an antiarrhythmic role through the negative regulation of miR-1 and its target genes mediated by sEHis.

### SEHis Regulate miR-1 by Acting on the PI3K/Akt/GSK3β Pathway

**Figure 2:**
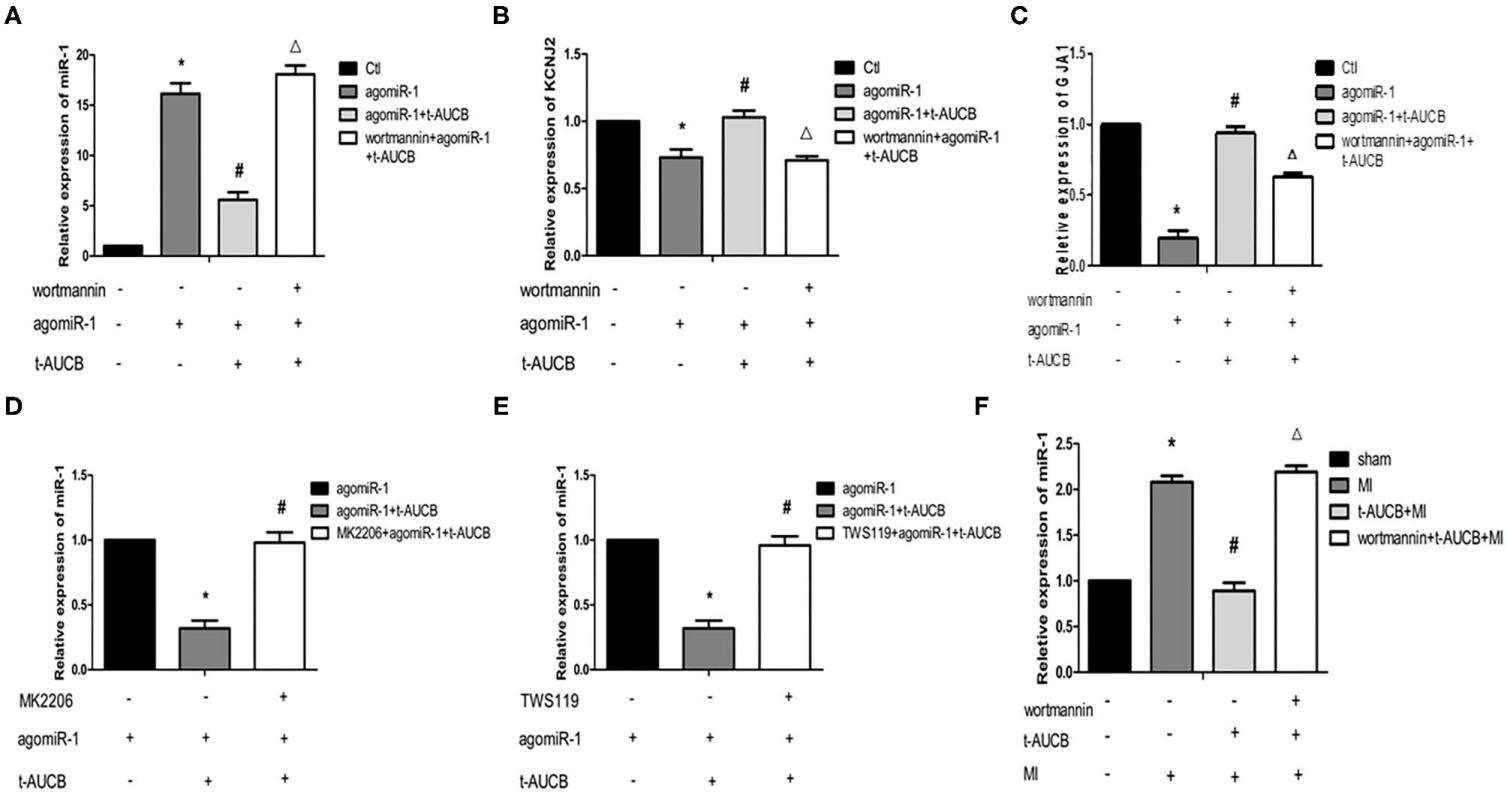
SEHis regulate miR-1 by acting on the PI3K/Akt/GSK3β pathway. PI3K, Akt, and GSK3β inhibitors, and t-AUCB were used to treat mouse cardiomyocytes overexpressing microRNA-1 (miR-1) and mice with myocardial infarction (MI). Real-time quantitative PCR tested the relative mRNA expressions of miR-1 and its target genes. **(A)** Wortmannin (inhibitor of PI3K) reversed the negative regulation of miR-1 by t-AUCB in neonatal mouse cardiomyocytes (*n* = 4). **(B)** Wortmannin reversed the effect of t-AUCB on KCNJ2 (*n* = 4). **(C)** Wortmannin reversed the effect of t-AUCB on GJA1 (*n* = 4). **(D)** MK2206 (inhibitor of Akt) inhibited the effect of t-AUCB on miR-1 (*n* = 6). **(E)** TWS119 (inhibitor of GSK3β) increased miR-1 levels (*n* = 6). **(F)** Effect of wortmannin on miR-1 in ischemic myocardium of mice after t-AUCB intervention (*n* = 4). Compared with the control (Ctl) group, *P < 0.05; compared with the agomiR-1 group, ^#^*P* and *P < 0.05; compared with the agomiR-1 + t-AUCB group, ^Δ^*P* and *P < 0.05.

The previous experimental results by our group showed that the levels of p-Akt and p-Gsk3β in the myocardial ischemic area in mice were significantly increased, and t-AUCB reversed these changes, suggesting that sEHis could activate the PI3K/Akt/Gsk3β pathway. Therefore, we investigated whether the PI3K/Akt/Gsk3β pathway was involved in the regulation of miR-1 by sEHis. *In vitro*, we blocked the PI3K/Akt/GSK3β pathway in neonatal mouse cardiomyocytes with PI3K, Akt, and GSK3β inhibitors. Compared with that in the agomiR-1 group, the expression of miR-1 in the agomiR-1 + t-AUCB group was significantly decreased (Figures 2A–E, *n* = 4 or *n* = 6, *P* < 0.05). However, wortmannin, an inhibitor of PI3K, increased miR-1 to 3.23-fold and decreased its target genes KCNJ2 and GJA1 to 69 and 67%, respectively (Figures 2A–C, *n* = 4, *P* < 0.05) compared with those in the agomiR-1 + t-AUCB group. MK2206 (an inhibitor of Akt) and TWS119 (an inhibitor of GSK3β) showed the same effects; these inhibitors also increased the expression of miR-1 to 3.08- and 3-fold, respectively (Figures 2D,E, *n* = 6, *P* < 0.05). *In vivo*, we further verified this conclusion by injecting wortmannin, a PI3K inhibitor, into the tail veins of mice. We found that compared with sham surgery, MI surgery increased the relative expression of miR-1, and t-AUCB pretreatment decreased the expression of miR-1 to 43% in mice with MI, but the effect was counteracted by wortmannin. The results showed that compared with that in the t-AUCB + MI group, miR-1 in the t-AUCB + MI + wortmannin group increased 2.46-fold (Figure 2F, *n* = 4, *P* < 0.05). These results demonstrated that sEHis regulated miR-1 through the PI3K/Akt/GSK3β pathway.

### The Effect of PI3K Subtypes on the Regulation of miR-1 by sEHis

**Figure 3:**
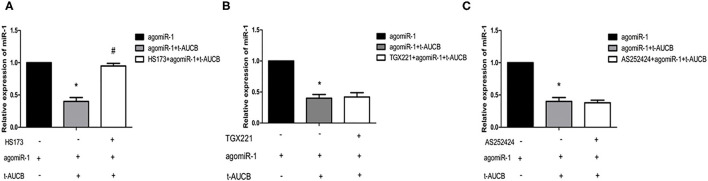
Effects of three PI3K subtypes on t-AUCB-mediated regulation of miR-1. Primary neonatal mouse cardiomyocytes were treated with PI3Kα inhibitor HS173, PI3Kβ inhibitor GX221, and PI3Kγ inhibitor AS252424 for 6 h, and then the cells were transfected with micrONTM mmu-miR-1a-3p croRNA-1 (agomiR-1), and treated with t-AUCB. The expression of miR-1 was detected. **(A)** T-AUCB significantly decreased miR-1 levels, and HS173 significantly reduced this effect. **(B)** GX221 was not involved in t-AUCB-mediated regulation of miR-1. **(C)** AS252424 were not involved in t-AUCB-mediated regulation of miR-1. Compared with the agomiR-1 group, *P < 0.05; compared with the agomiR-1 + t-AUCB group, ^#^*P* < 0.05; all *n* = 6.

PI3K has four subtypes (PI3Kα, PI3Kβ, PI3Kγ, and PI3Kδ), and the catalytic subunit of PI3Kδ only exists in leukocytes. To investigate the effects of different subunits on sEHi-mediated regulation of miR-1, primary neonatal mouse cardiomyocytes were treated with the PI3Kα inhibitor HS173, PI3Kβ inhibitor GX221, and PI3Kγ inhibitor AS252424 for 6 h. Then, the cells were transfected with agomiR-1 and treated with t-AUCB. The results showed that compared with that in the agomiR-1 group, miR-1 in the agomiR-1 + t-AUCB group decreased to 40% (Figures 3A–C, *n* = 6, *P* < 0.05). However, miR-1 expression in HS173-pretreated cells increased 2.37-fold when compared with that in the agomiR-1 + t-AUCB group (Figure 3A, *n* = 6, *P* < 0.05), while miR-1 in GX221- and AS252424-pretreated cells increased by 1.05-fold and 95%, respectively (Figures 3B,C, *n* = 6, *P* > 0.05). Therefore, we believe that only PI3Kα is involved in the effect of t-AUCB on miR-1.

### The Interaction Between SRF and PI3K/Akt/GSK3β Pathway in sEHi-Mediated Regulation of miR-1

#### Effects of t-AUCB on Myocardial Tissue SRF Protein in the Ischemic Area of Mice After MI After PI3K Inhibition

**Figure 4:**
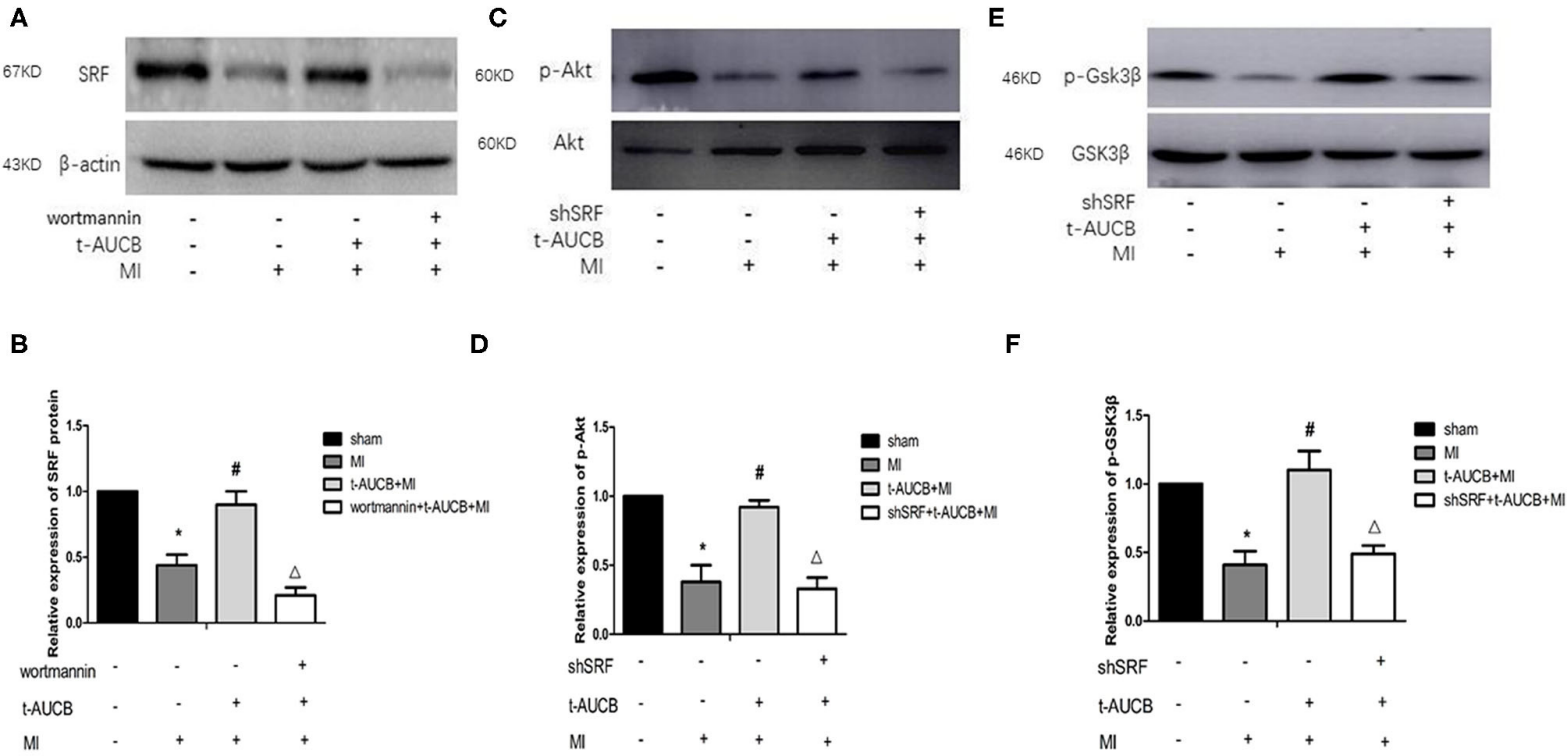
The interaction between SRF and PI3K/Akt/GSK3β pathway during the regulation of miR-1 by sEHis. One week after intervention with t-AUCB in mice with myocardial infarction (MI), PI3K inhibitor wortmannin was injected into the tail vein, then serum response factor (SRF) was detected; on the other hand, MI mice were injected with effective SRF small hairpin RNA sequence adenovirus (shSRF) and treated with t-AUCB, the expressions of p-Akt and p-GSK3β were detected by western blot. **(A)** After blocking the PI3K pathway, the effects of t-AUCB on SRF were examined. **(B)** Relative protein expression of SRF after the treatment of wortmannin. **(C)** After silencing SRF, the effects of t-AUCB on Akt and p-Akt were examined. **(D)** Relative protein expression of Akt and p-Akt. **(E)** After silencing SRF, the effects of t-AUCB on GSK3β and p-GSK3β were examined. **(F)** Relative protein expression of GSK3β and p-GSK3β. Compared with the sham group, *P < 0.05, compared with the MI group, ^#^*P* < 0.05, compared with the t-AUCB+MI group, ^Δ^*P* < 0.05; all *n* = 5.

To further clarify the mechanism by which sEHis regulate SRF, we used western blotting to examine the effects of t-AUCB on the protein expression of SRF in the myocardial ischemic area in mice with MI after PI3K inhibition. MI decreased the protein expression of SRF, while t-AUCB significantly increased it. After the t-AUCB intervention, wortmannin, a PI3K inhibitor, abrogated the effect of t-AUCB on SRF (Figures 4A,B, n = 5, *P* < 0.05). These findings also suggested that the PI3K pathway was involved in the positive regulation of SRF by t-AUCB.

#### Effects of t-AUCB on p-Akt and p-GSK3β in Myocardial Tissue in Mice After MI and SRF Silencing

To determine whether SRF modulated the effects of t-AUCB on the PI3K/Akt/GSK3β pathway, we measured the expression of p-Akt and p-GSK3β after silencing SRF with shSRF. The results showed that p-Akt and p-GSK3β in the shSRF + t-AUCB + MI group were significantly decreased compared with those in the t-AUCB + MI group, which were 36 and 45% of their levels before silencing, respectively (Figures 4C–F, *n* = 5, *P* < 0.05). We concluded that silencing SRF antagonized the increases in Akt and GSK3β phosphorylation caused by t-AUCB. These results showed that SRF was involved in the t-AUCB-mediated regulation of the PI3K/Akt/GSK3β pathway.

## Discussion

**Figure 5:**
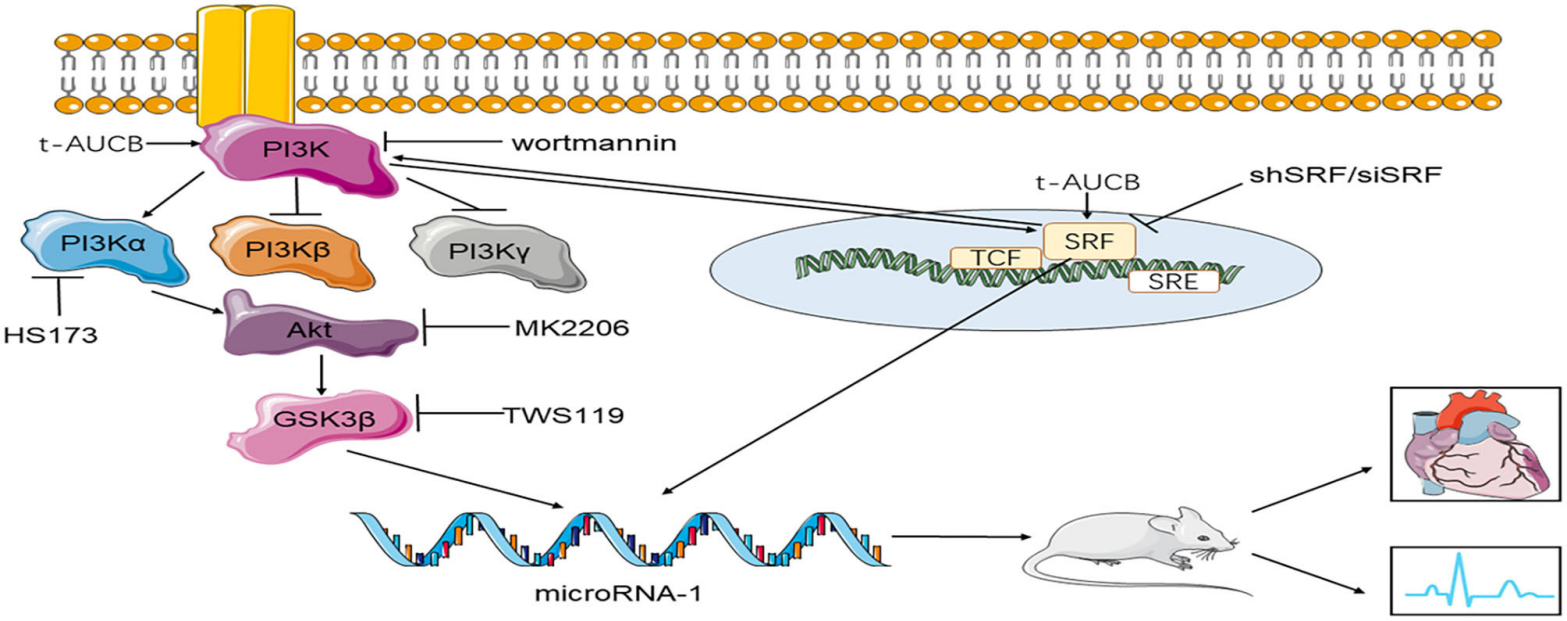
The mechanism of t-AUCB regulation of ischemic arrhythmia. T-AUCB, a preparation of sEHis, regulates microRNA-1 by regulating SRF and the PI3Kα/Akt/GSK3β pathway, thereby reducing the size of myocardial infarction and inhibiting ischemic arrhythmias. During this process, the PI3Kα/Akt/GSK3β pathway and SRF interact with each other. SRF indicates serum response factor; shSRF indicates SRF small hairpin; siSRF indicates SRF small interfering RNA; wortmannin is the inhibitor of PI3K; HS173 indicates PI3Kα inhibitor; MK2206 is the inhibitor of Akt; TWS119 is the inhibitor of GSK3β.

Our study showed for the first time that sEHi t-AUCB regulated miR-1 and its target genes by regulating SRF expression and the PI3K/Akt/GSK3β pathway, thereby inhibiting ischemic arrhythmias. During this process, the PI3K/Akt/GSK3β pathway was involved in the positive regulation of SRF by t-AUCB, and SRF positively regulated the effects of t-AUCB on Akt and Gsk3β phosphorylation. Both factors influenced each other (Figure 5).

The treatment of ischemic arrhythmia is a clinical hotspot and a challenge in recent years. A large number of studies have shown that miR-1 has arrhythmic effects (Costantini et al., 2005; Yang et al., 2007; Zhao et al., 2007; Liu et al., 2017; Guo et al., 2020). Our previous study showed that sEHis negatively regulated miR-1. Furthermore, we also found that the protein expression of SRF in the ischemic area in mice with MI was significantly reduced, and the preparation of sEHis reversed this change. Therefore, we hypothesized that sEHis could regulate the expression of miR-1 by affecting SRF. To clarify this hypothesis, we knocked down SRF in a neonatal mouse myocardial model that overexpressed miR-1 and interfered with t-AUCB. We found that miR-1 expression increased and that the negative regulation of miR-1 and the positive effect on its target genes caused by sEHis were weakened, and we further confirmed these research results *in vivo*. These outcomes all indicated that SRF could negatively regulate the expression of miR-1, and sEHis negatively affected the expression of miR-1 by increasing the level of SRF. Pan et al. (2016) showed that miR-1 was obviously increased, and SRF protein was decreased in skeletal muscle cells that were intermittently exposed to hypoxia and hypercapnia, and these changes were further amplified after electrical stimulation. A study by Zhang et al. (2011) demonstrated that the expression of miR-1 was reduced in the mouse heart after SRF overexpression and increased when SRF was reduced. Moreover, Jiang et al. (2010) also suggested that SRF directly induced the transcription of miR-1 in cardiac and skeletal muscle precursor cells. These results were all consistent with our study, suggesting that SRF had a negative role in regulating miR-1 expression. Some studies have suggested that SRF works by binding to the serum response element, which contains the response element GArG (Treisman, 1986; Boxer et al., 1989; Miano, 2003), and two GArG-like elements have been found in the promoter of the miR-1 gene (Zhao et al., 2005). Therefore, researchers believe that SRF might directly act on miR-1. This conclusion further provided a theoretical basis for the results of our study.

However, Lu et al. (2009) found that the expression of miR-1 and SRF in ischemic myocardial tissues of rats was significantly increased when studying the mechanism by which propranolol regulates miR-1, and after propranolol pretreatment, miR-1 and SRF were significantly reduced. Shan et al. (2009) also showed that miR-1 and SRF in myocardial tissue were increased 3 months after MI, and tanshinone treatment reversed these changes. These results suggested that SRF and miR-1 were positively correlated, which was contrary to the results of our study. The inconsistency might be related to the different time points of specimen collection and differences in the intervention drugs. The specific mechanism needs to be further studied.

A number of studies have confirmed that activation of the PI3K/Akt/GSK3β pathway plays an important protective role in preventing myocardial ischemia. Zhu et al. (2006) studied the myocardial protection mechanism of ischemic post-processing and found that myocardial protection was achieved by activating the PI3K pathway. Cao et al. (2005) found that methionine-enkephalin exerted a protective effect on cardiomyocytes through the PI3K pathway in a model of isolated adult rabbit cardiomyocytes simulated by myocardial ischemia. Our previous experiments showed that t-AUCB reversed the reductions in p-Akt and p-Gsk3β levels in the ischemic myocardium of mice, suggesting that sEHis perhaps activated the PI3K/Akt/GSK3β pathway (Gui et al., 2017). Therefore, in this study, the PI3K inhibitors, wortmannin and t-AUCB, were administered to neonatal cardiomyocytes overexpressing miR-1. The results showed that wortmannin significantly reduced the effect of sEHis on miR-1 and its target genes. To further verify these results *in vivo*, we injected 8-week-old male mice with wortmannin through the tail vein and established an MI model after t-AUCB pretreatment. The results showed that the negative regulation of miR-1 by sEHis was weakened. These *in vitro* and *in vivo* findings all suggested that sEHis negatively affect the expression of miR-1 by activating PI3K/Akt/GSK3β pathway. There have been few studies about PI3K-mediated regulation of miR-1 for myocardial protection. When Hao et al. (2018) explored the effect of miR-1 on myocardial ischemia-reperfusion injury in rats pretreated with sevoflurane, he found that miR-1 was expressed at high levels in rats with ischemia-reperfusion, while MAPK3 was expressed at low levels, and excessive miR-1 expression or blockade of the PI3K pathway caused by silencing MAPK3 increased cell apoptosis, ischemia risk or infarct zone, and the concentration of lactate dehydrogenase. Chen et al. (2012) showed that insulin reduced miR-1 and had a significant protective effect on the damage brought about by miR-1 under oxidative stress. He thought that this phenomenon was mediated by the PI3K pathway, and that it might be one of the principles of the cardiovascular effects of insulin. These results were consistent with our study.

Akt is a serine-threonine kinase that is activated by PI3K (Parsa et al., 2003; Tramontano et al., 2003). This study showed that Akt inhibitors restrained sEHi-mediated regulation of miR-1. Experiments by Raphael et al. (2006) showed that increasing Akt phosphorylation inhibited myocardial ischemia, and studies published in Nature Medicine showed that Akt phosphorylation negatively affected miR-1 expression (Care et al., 2007). The results of this study and others pointed out that sEHis might negatively adjust miR-1 expression by increasing the phosphorylation level of Akt, thereby exerting an antiarrhythmic effect.

There are currently no studies on Gsk3β and miR-1. Some researchers insisted that the cardioprotective effect of Gsk3β was achieved by the phosphorylation of Gsk3β, which inhibited itself, and Akt phosphorylation induced the phosphorylation of Gsk3β (Juhaszova et al., 2009; Wu et al., 2012). We found that pretreatment of neonatal mouse cardiomyocytes with a GSK3β inhibitor reversed the effects of sEHis on miR-1 levels in an miR-1 overexpression cell model, which further confirmed that sEHis played a role through the PI3K/Akt/GSK3β pathway.

In addition, a study showed that EETs extenuated insulin resistance via the PI3K/Akt signaling pathway in cultured bovine aortic endothelial cells (Wang et al., 2003), and Dhanasekaran et al. (2008) suggested that EETs were able to activate numerous targets of PI3K/Akt in a model of hypoxia/reoxygenation in primary cultured cardiomyocytes, such as an increase in PI3K activity and Akt phosphorylation. Therefore, we hypothesized that EETs exert their cardioprotective effects in ischemic and hypoxic environments through PI3K/Akt/GSK3β pathway. *In vitro* and *in vivo* experiments showed that sEHis positively affected the expression of miR-1 by activating PI3K/Akt/GSK3β pathway to induce cardioprotective effects, which verified this hypothesis.

More interestingly, during the experiment, we also found that PI3Kα inhibitors reversed the effects of sEHis on miR-1 levels in primary cultured mouse cardiomyocytes, while PI3Kβ and PI3Kγ inhibitors had no such effect, suggesting that the specific target through which sEHis regulate miR-1 through the PI3K/Akt/GSK3β pathway was PI3Kα. Lu et al. (2012, 2013) found that cardiomyocyte APD was prolonged in mice whose PI3Kα catalytic subunit gene was knocked out, while continuous activation of the PI3Kα catalytic subunit shortened the APD of myocardial cells. Yang et al. (2010) also showed that activation of the PI3Kα signaling pathway in pathological hypertrophic cardiomyocytes maintained normal myocardial cell electrical activity, which in turn prevented arrhythmias in pathological cardiomyopathy and reduced the risk of sudden death. Overall, this study confirmed that PI3Kα played a dominant role in the negative regulation of miR-1 by sEHis through the PI3K/Akt/GSK3β pathway.

As described previously, the effects of sEHis on miR-1 were achieved by positively regulating SRF protein expression and activating the PI3K/Akt/GSK3β pathway. Did the PI3K/Akt/GSK3β pathway and SRF affect each other during this process? For further verification, we established a mouse model of MI and treated the mice with shSRF and wortmannin. The results showed that sEHis positively regulated the levels of SRF, p-Akt, and p-Gsk3β in the myocardial ischemic area, while wortmannin and shSRF attenuated these changes, indicating that SRF and PI3K were positively correlated in the process by which sEHis regulated miR-1.

There was no final correlation between SRF and PI3K in various biological effects. Small (Small et al., 2010) indicated that miR-486 was directly affected by SRF in skeletal muscle cells, and the activity of the PI3K/Akt signaling pathway was increased when miR-486 was overexpressed, indicating that miR-486 was downstream of SRF and could also affect PI3K/Akt signaling pathway activity. Mutlu et al. (2016) also showed that miR-564 inhibited the activity of PI3K by negatively regulating the mRNA and protein expression of SRF. These experimental results were consistent with the present study; that is, there was a positive correlation between SRF and PI3K. On the other hand, some researchers found that carcinogens abnormally activated the PI3K signaling pathway and reduced the binding of SRF to its site, and inhibiting the PI3K signaling pathway restored the level of SRF in cells (Shin et al., 2006), suggesting that PI3K negatively regulated SRF protein levels. This result was completely opposite to our findings, so the correlation between SRF and PI3K/Akt/GSK3β pathway and its specific mechanism are worthy of further examination. However, PI3K was clearly involved in the positive regulation of SRF protein levels, and SRF also participated in the activation of the PI3K pathway caused by sEHis. The PI3K pathway interacts with SRF in the negative regulation of miR-1 by sEHis.

## Conclusion

Both the SRF and the PI3Kα/Akt/GSK3β pathway are involved in the negative regulation of miR-1 by t-AUCB, and these factors interact with each other in this process. These results provide a new strategy and theoretical basis for the clinical treatment of ischemic arrhythmia.

## Limitations

This study has certain limitations. Although it has been verified that sEHis can protect the ischemic myocardium of mice with MI through the SRF and the PI3Kα/Akt/GSK3β pathway, thereby reducing the occurrence of an ischemic arrhythmia, we did not measure the action potential duration, current density, or other related indicators of arrhythmia in the overall animal experiment. Therefore, it is necessary to further examine these indicators and explore the mechanism by which sEHis affect ischemic arrhythmia.

## Data Availability Statement

The original contributions presented in the study are included in the article/supplementary material, further inquiries can be directed to the corresponding author/s.

## Ethics Statement

The animal study was reviewed and approved by the Institutional Animal Care and Use Committee of the Second Xiangya Hospital of Central South University, and the experiments were in accordance with the NIH Guide for the care and use of Laboratory Animals.

## Author Contributions

DX experimental design. YC and QL experimental performance. YC, QL, and TY data analysis. LS and DX manuscript revision. YC manuscript writing. DX funding acquisition. All authors contributed to the article and approved the submitted version.

## Funding

The authors disclosed receipt of the following financial supports for the research, authorship, and/or publication of this article: this work was supported by National Natural Science Foundation of China (Nos. 81871858, 82172550).

## Conflict of Interest

The authors declare that the research was conducted in the absence of any commercial or financial relationships that could be construed as a potential conflict of interest.

## Publisher's Note

All claims expressed in this article are solely those of the authors and do not necessarily represent those of their affiliated organizations, or those of the publisher, the editors and the reviewers. Any product that may be evaluated in this article, or claim that may be made by its manufacturer, is not guaranteed or endorsed by the publisher.
